# Malignant juxtaglomerular cell tumor

**DOI:** 10.1016/j.eucr.2022.102176

**Published:** 2022-07-31

**Authors:** Daniel Geisler, Fawaz Almutairi, Ivy John, Gabriela Quiroga-Garza, Michelle Yu, Raja Seethala, Sheldon Bastacky

**Affiliations:** a Department of Pathology, University of Pittsburgh Medical Center, Pittsburgh, PA, USA; b Department of Urology, University of Pittsburgh Medical Center, Pittsburgh, PA, USA

**Keywords:** Kidney, Juxtaglomerular cell tumor, Malignant, Renin, GATA3

## Abstract

Juxtaglomerular cell tumors (JGCTs) are rare, typically benign neoplasms; only rare cases are clinically or histologically malignant. We herein report the histologic, immunophenotypic, and molecular features of a clinically unsuspected, diagnostically challenging case of malignant JGCT in a 23-year-old man. The diagnosis is confirmed with electron microscopy. The case is notable for its marked mitotic activity, which has not been previously reported in JGCTs, and novel finding of GATA3 immunohistochemical positivity.

## 1 Introduction

Juxtaglomerular cell tumors (JGCTs) are rare, typically benign neoplasms; only rare cases are clinically or histologically malignant. We herein report the histologic, immunophenotypic, and molecular features of a clinically unsuspected, diagnostically challenging case of malignant JGCT.

## 2 Case presentation

**Fig. 1:**
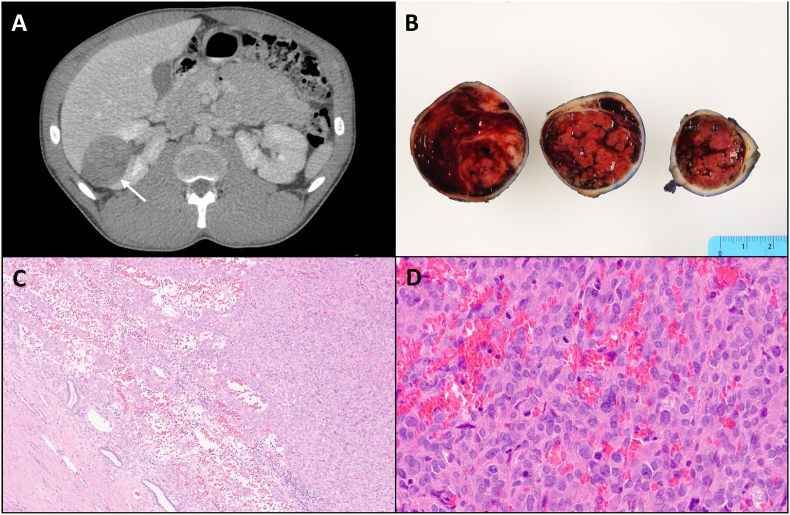
An axial computed tomography image with intravenous contrast demonstrating a solid mass (white arrow) in the lateral mid pole of the right kidney (**A**). Gross image of the tumor showing well-defined borders with a fleshy, hemorrhagic cut surface (**B**). Tumor borders are relatively sharp with occasional entrapped renal tubules at the periphery (**C**). The tumor is composed of ovoid to spindle cells with moderate nuclear pleomorphism, predominantly eosinophilic cytoplasm, and intimately associated with surrounding delicate vasculature. Mitotic figures are readily apparent (**D**).

**Fig. 2:**
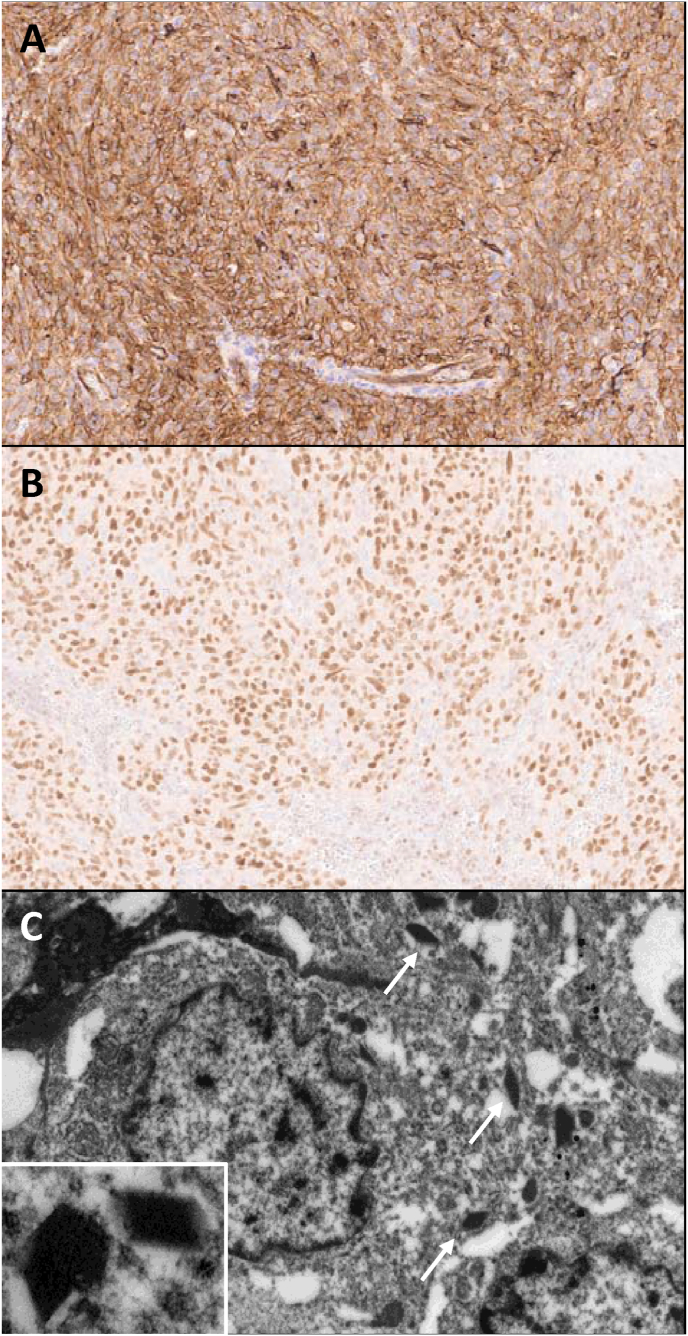
Immunohistochemical stains show tumor cells are diffusely positive for CD34 (**A**) and GATA3 (**B**). Electron microscopy shows tumor cells with cytoplasmic electron-dense rhomboid renin protogranule crystals (white arrows, inset) (**C**).

A 23-year-old man was incidentally found to have hypertension (162/96 mmHG). Other than a mildly low potassium (3.4 mmol/L), a basic metabolic panel was unremarkable. Abdominal ultrasound and computed-tomography were performed and identified a 4.8 cm solid-appearing, well-marginated mass in the right kidney (Fig. 1A). The patient underwent robotic-assisted partial nephrectomy to remove the mass. The gross examination of a partial nephrectomy specimen showed an enucleated lesion with a glistening, fleshy, and hemorrhagic cut surface (Fig. 1B). Microscopic examination showed a circumscribed spindle cell neoplasm with ovoid central nuclei, small nucleoli, and eosinophilic to focally clear cytoplasm. The tumor was closely associated with a network of thin and thick walled vasculature (Fig. 1C and D). In particular, moderate nuclear pleomorphism and marked mitotic activity was seen with up to 12 mitoses per 2 mm^2^ identified. No necrosis or vascular invasion was identified. A panel of immunohistochemical stains showed the neoplasm was diffusely positive for CD34 (Fig. 2A) and GATA3 (Fig. 2B), with weak positivity for synaptophysin. AE1/AE3, CAM5.2, EMA, chromogranin, CD56, S100, SOX10, HMB45, cathepsin K, CD31, ERG, desmin, smooth muscle actin, DOG1, CD117, CD99, FLI1, PAX-8, TLE1, STAT6, Beta-catenin and ALK1 were negative. Succinyl dehydrogenase (SDHB) and INI1 protein expression was preserved.

A next generation sequencing (NGS) sarcoma fusion panel (Sarcoma Fusion NGS Panel, MiSeq; Cleveland Clinic, Cleveland, OH, USA) showed no gene fusions. In addition, targeted NGS analysis (Oncomine, Ion Torrent; UPMC, Pittsburgh, PA, USA) showed no tier I/II ACMG/AMP DNA mutational variants, gene fusions, or copy number alterations. However, electron microscopy revealed rhomboid, electron-dense renin protogranules in neoplastic cells (Fig. 2C) and a diagnosis of malignant JGCT was rendered. The patient's hypertension and hypokalemia resolved post resection, and close clinical follow-up is planned with radiologic surveillance at 3 mothns post resection.

## 3 Discussion

JGCTs, or reninomas, arise from the modified smooth muscle cells in the juxtaglomerular apparatus. JGCTs present predominantly in young adults, with a peak incidence in the second and third decades of life, and show a slight female predominance.^1^ Clinically, JGCTs are classified into three types: typical, atypical and nonfunctioning.^2^ Typical JGCTs present with high renin levels and hypertension, hypokalemia, and hyperaldosteronism. Atypical JGCTs present with hypertension and show normal potassium levels, while nonfunctioning JGCTs present with normal blood pressure and potassium levels.

The aforementioned gross, microscopic, and immunohistochemical features are fairly typical of previously reported JGCT. Though not present here, variable muscle marker expression is often noted.^1^ However, while classic JGCTs exhibit mild to moderate cytologic atypia without significant mitotic activity or abnormal mitotic figures,^3^^,^^4^ this case demonstrated overtly malignant histologic features. Their rarity already makes JGCTs diagnostically challenging, and malignant features add further to this challenge, raising additional differential diagnoses that span sarcomas, carcinomas, and even neuroendocrine neoplasms. Clinical context, recognition of more typical JGCT morphology, and the distinctive immunophenotype may help exclude other considerations. Immunohistochemical or electron microscopy identification of renin protogranules, if available, would also be pathognomic for JCGT.

Interestingly, JGCTs show some morphologic and immunohistochemical overlap with glomus tumors, and given the presumed origin from the juxtaglomerular apparatus, it is appealing to postulate a relationship with tumors that show “true” pericytic differentiation. Strong, diffuse expression of CD34 may favor JGCTs over glomus tumor, however, the specificity is questionable due to the rarity of renal glomus tumors.^3^ On the other hand, our case shows diffuse positivity for GATA3, which has been previously documented in only one other report.^5^ Notably, an additional case of benign JCGT from our institutional archives also showed diffuse GATA3 positivity, suggesting diagnostic utility. Literature on the genetic abnormalities of JGCTs is limited, and targeted NGS in the current case did not detect any pathologic molecular alterations, including *NOTCH* fusions characteristically seen in glomus tumors. The absence of common fusions and mutations suggests a unique pathogenesis from glomus tumor (and other pericytomatous tumors).

At least three malignant JCGTs have been published with common reported clinical features including increased size (greater than 8 cm in largest dimension) and advanced age at time of diagnosis. Common reported histologic features included lymphovascular invasion, marked cytologic atypia, and a mildly increased mitotic rate. On clinical follow-up, two cases developed distant metastasis and one case showed local recurrence after one year.^3^

## 4 Conclusion

In conclusion, we report a rare case of malignant JCGT with the novel finding of GATA3 positivity, which may help resolve differential diagnostic considerations. Despite application of available NGS panels, a driver mutation remains elusive.

## Funding

This research did not receive any specific grant from funding agencies in the public, commercial, or not-for-profit sectors.

## Contributions

DG, FA, IJ, GQG, RS, SB contributed with investigation, formal analysis, resources, review, and editing. DG and FA also contributed with original draft writing.

## Declaration of competing interest

The authors have no conflicts of interest to disclose.
